# Initial evaluation of the "Trauma surgery course"

**DOI:** 10.1186/1749-7922-1-5

**Published:** 2006-03-24

**Authors:** Gregorio Tugnoli, Sergio Ribaldi, Marco Casali, Stefano M Calderale, Massimo Coletti, Marco Alifano, Sergio N Forti Parri, Silvia Villani, Andrea Biscardi, M Chiara Giordano, Franco Baldoni

**Affiliations:** 1 Chirurgia d'Urgenza e del Trauma (Head: Dr. Franco Baldoni), Dipartimento Emergenza, Ospedale Maggiore, L.go Nigrisoli 2, 40133 Bologna, Italy; 2 Gruppo Aperto per lo Studio del Trauma (GAST), Clinica Chirurgica d'Urgenza e di Pronto Soccorso, Università La Sapienza, Roma, viale del Policlinico 2, 00100 Roma, Italy; 3 Chirurgia Toracica (Head: Dr. Maurizio Boaron), Ospedale Maggiore, L.go Nigrisoli 2, 40133 Bologna, Italy

## Abstract

**Background:**

The consequence of the low rate of penetrating injuries in Europe and the increase in non-operative management of blunt trauma is a decrease in surgeons' confidence in managing traumatic injuries has led to the need for new didactic tools. The aim of this retrospective study was to present the Corso di Chirurgia del Politrauma (Trauma Surgery Course), developed as a model for teaching operative trauma techniques, and assess its efficacy.

**Method:**

the two-day course consisted of theoretical lectures and practical experience on large-sized swine. Data of the first 126 participants were collected and analyzed.

**Results:**

All of the 126 general surgeons who had participated in the course judged it to be an efficient model to improve knowledge about the surgical treatment of trauma.

**Conclusion:**

A two-day course, focusing on trauma surgery, with lectures and life-like operation situations, represents a model for simulated training and can be useful to improve surgeons' confidence in managing trauma patients. Cooperation between organizers of similar initiatives would be beneficial and could lead to standardizing and improving such courses.

## Background

The treatment of the thoraco-abdominal trauma has always represented a surgical challenge, owing to the peculiarity of these injuries. The need for specific training for surgeons involved in the care of these patients is justified by the difficulties in obtaining an exhaustive pre-operative assessment, the need for prompt decision-making, and the often limited available resources.

Furthermore, the number of surgically treated trauma patients has markedly decreased in recent years, owing to many factors, such as the low rate of penetrating trauma, the improved safety system of vehicles, innovations in diagnostic tools, and the discovery of alternative treatments [1-4]. Thus, all these factors have prompted a discussion about the future of trauma surgeon training [5,6].

A variety of didactic methods, based on "simulated training", have been suggested [7,8] and computer simulation or computer-controlled dummies have been employed. Another proposed training method involves practice on cadavers or animal models. The use of *in vivo *animal models, generally large-sized swine, which simulate human thorax and abdomen quite well, enables extremely realistic situations to be recreated, even to the point of putting the participants under stress [7,9,10].

In Italy the general surgeon is trained by six-year residency in General Surgery or in General and Emergency Surgery. However, there are no specific residency or university courses for Trauma Surgery. Managing trauma differs greatly from region to region; in most regions there is a lack of reference centers for thoracic and abdominal traumas, which are treated by the general surgeon of the nearest hospital, whereas specific traumas, such as neurosurgical, orthopedic and burns, are treated by specialized surgeons.

Instead, in our region (Emilia-Romagna) there are three reference trauma center (Parma, Bologna, Cesena). These centers are equipped with all the necessary resources to treat all kinds of trauma.

Since November 2002, a multi-trauma surgery-training course has been running in Bologna, Italy, which is mainly aimed at general surgeons, who, owing to their work, more frequently run into trauma injuries and residents who currently have less chance to gain experience in trauma surgery.

The aim of this study was to present this course and discuss its purposes and educational effectiveness compared with similar courses. We also wanted to assess whether our model could be used as a qualified updating course. We present herein the results obtained in the first seven editions of the course.

## Materials and methods

Our project is based on the experience acquired in thoracic and abdominal injuries at Maggiore Hospital of Bologna, Italy, over the last 16 years. This hospital has been a reference centre for trauma management for several years, with over 400 cases of major (ISS>25) traumas per year. Cooperation has been set up between our multidisciplinary team of Emergency Surgery and Trauma and the Open Group for the Study of Trauma (*Gruppo Aperto per lo Studio del Trauma – GAST*) belonging to the Clinical Emergency Surgery and Emergency Unit at La Sapienza University in Rome, Italy. The Trauma Surgery Course was conceived to share the experience of these two groups with other surgeons involved in trauma patient management. The course is mainly aimed at thoracic and abdominal trauma, as this usually involves the general surgeon, while specific topics, such as neurosurgical or orthopedic aspects are addressed in lessons on "Multispecialized approach in E.R." and "Complex pelvic truma".

All the teaching staff, consisting of 17 healthworkers, have a long specific experience in trauma treatment, each in their own particular field (11 general surgeons, 2 intensive care physicians, 1 radiologist, and 3 registered nurses of the ward and operating room of the trauma center); 12 out of 14 physicians are ATLS qualified, and 8 of them are ATLS instructors.

The course is restricted to 18 participants, selected on a first come first served basis, owing to the difficulty to equip the veterinary operating rooms and enable each participant to practice the surgical techniques. The course lasts two days. Day one focuses on theoretical aspects, by analyzing the main topics of diagnosis and treatment (surgery, interventional radiology, and resuscitation) (Table 1) of polytrauma, using slides and video presentations. Moreover, a simulated clinical case is presented and discussed to bring together all the topics and also enables the introduction of interactive debate on pre-operative management, optimization, and decision making.

**Table 1:** Topics dealt with during the theoretic part of the course

Multispecialized approach in E.R.	Thorax surgery
Diagnosis by imaging	Liver Surgery
Emergency thoracotomy	Indications for Transplantation in liver trauma
Abdomen Surgery	Pancreas and Duodenal Surgery
Damage Control Surgery	Spleen Surgery
Complex pelvic trauma	Face trauma
Polytrauma Resuscitation treatment	Risk Management in the operating room
Hollow organ surgery	Neck surgery
Compartmental Abdominal syndrome	Management of operating room
Nursing in polytrauma	

The second day is held in the Institute of Veterinary-Medicine (Clinical Veterinary Department – Surgery Unit) and consists of surgical exercise on large-sized swine. All the participants are invited to perform the common surgical procedures (Table 2) used in the management of trauma and are requested to treat the injuries produced by the tutor, who simulates a clinical case. During this stage all the techniques (tracheostomy, intubation, chest tube) the trauma surgeon should be familiar with are covered.

**Table 2:** Surgical techniques performed by the participants during the practical section of the course

Abdominal lesion recognition	Liver parenchymal suture
Abdominal aorta vascular clamping	Debridement
Spleen vascular exploration and control	Atypical resection of liver
Splenic parenchymal suture	Liver Packing
Spleen partial resection	Thoracotomy and suture of intercostals vessels
Splenectomy	Thoracoabdominal aortic clamping
Renal exploration and vascular control	Pulmonary parenchymal repair/resection
Kidney parenchymal suture	Main bronchus Suture
Partial kidney resection	Isolation and vascular control of pulmonary hilus
Nephrectomy	Treatment of pericardial tamponade
Biliary tract evaluation	Heart edge suture

The importance of the effectiveness and speed of intervention is frequently stressed. The animals are alive, intubated and monitored under the care of a veterinary anesthetist for the duration of the intervention. To better simulate a life-like situation, each table of the operating theatre is equipped and served by a qualified surgery nurse. For each animal there are 3 participants, distributed according to their capability and experience, and 2 tutors (1 for the abdomen and 1 for the thorax). The animals are treated in accordance with the Italian law on the use of laboratory animals.

The comprehensive evaluation of the degree of learning takes into account technical skills, the ability to understand the clinical aspects, identify priorities, and repair the induced lesions in a life-like situation. The participants are evaluated by the tutors of the practical part of the course and rated: insufficient, sufficient, good, and excellent. Three different scores are attributed for abdominal surgical techniques, thoracic surgical techniques, and emergency surgery techniques, such as damage control and emergency thoracotomy.

Twenty CME credits (Continuous Medical Education) are awarded by the National Committee for Continuous Training. All the participants receive a form to fill in to evaluate the training course in terms of relevance of the topics, quality of teaching, and effectiveness in providing a continuous education.

The course is held twice a year and is standardized and fully reproducible.

At the end of course each participant is issued with a certificate.

## Results

In the first 7 courses held from 2002 to 2005, 126 participants attended from 19 regions, uniformly distributed among northern, central and southern Italy.

The mean age of participants was 43.9 years (range 30–60); ten were women (7,9%). 124 were general surgeons or worked for humanitarian organizations and 2 were fifth year residents. The mean work experience, after residency, was 16 years (range 3 months – 33 years). Nine (7.1%) participants were heads of surgery units.

All the participants attended the theoretical lessons and had the opportunity to practice the programmed surgical techniques starting from the abdomen and ending with the thorax and heart.

Since the participants were grouped based on their capability and experience, during the practical stage they were evaluated accordingly.

None of the participants received an "insufficient" score. (if a participant failed to get a sufficient score, the course could be repeated free of charge), with the majority of them rating good or excellent in all three fields (abdominal, thoracic, and emergency surgery techniques).

54 (42.8%) participants defined the course as "highly relevant", 70 (55.5%) "relevant", and 2 (1.5%) "quite relevant". None of the participants defined the course as "slightly" or "not relevant".

The quality of the teaching on the course was considered by the participants as "excellent", "good", and "satisfactory" in 81 (64,2%), 44 (34,9%), and 1 (0,7%) cases, respectively. In no instance the quality of teaching was considered as "mediocre" or "insufficient".

Finally, with respect to the evaluation of the efficacy of the course in providing a continuous education, the answers of participants were "highly efficacious", "efficacious", and "moderately efficacious" in 77 (61,1%), 36 (28,5%), and 13 (10,3%) cases, respectively, whereas none of the participants defined it "partially efficacious" or "totally inefficacious".

## Discussion

The need to train surgeons to apply emergency surgery techniques not commonly applied, and to use new instrumentation has led to the spread of training programs based on life-like simulations, using instrumental or animal models [11-14]. Normally, the participants attending the courses are post-graduate students of surgery, or general surgeons, who have no familiarity with the management of trauma patients.

The problem concerning specific training for the trauma surgeon has been dealt with in the past years by organizing courses with surgical simulation. The International Association for Trauma Surgery and Intensive Care organized a two-day course on the Definitive Surgical Trauma Care, including both theoretical and practical training on cadavers and live animals [15]. A one-day course focusing on the lesions of penetrating trauma was organized at Hartford, Connecticut, (ATOM, Advanced Trauma Operative Management) and was divided into a theoretical section and a practical part involving surgical training on large-sized swine. The details of the course and the results obtained from the first 50 participants have been recently published [9].

Though we started from a different background, our proposal is very similar. The structure of our course is original but comparable to the few courses held in the USA and in other non-European countries. The "main topics" and the life-like situations for the evaluation of practical skills are essentially the same. However, the peculiarity of our course is that we pay more attention to the multidisciplinary approach as well as to diagnostic and resuscitation problems. Another peculiarity of our course is the presentation of some lectures by experienced nurses, in the idea of providing supportive arguments to the concept that the traumatized patient is a very complex one, and that only a multidisciplinary approach can produce the best outcome.

We obtained very encouraging results from the first courses due to the high degree of attention paid during teaching sessions and the participation in the discussion of clinical cases. Therefore, although the parameters were not easily quantifiable, all the participants demonstrated with varying degrees of skill that they could successfully manage "unfamiliar surgical situations". Moreover, the participants judged the course to be very useful for their own training: more precisely, 98.3% rated the course favorably with regards to the need for personal updating; 99.1% for the quality of the teaching and 89,6% for the efficacy of the course for personal training. To further improve the theoretical aspects of the course, we currently mail some of the lectures before the course begins, with the aim of giving the participants pre-course preparation.

On this basis, we can reasonably assume that the course was successful, due both to the peculiarity of the topic and the involvement in the practical section of the course. The participants coming from all the Italian regions indicated that the need for CME for surgeons involved in the management of such injuries was felt in many centers.

Of note, all but two of the participants had previous vast working experience. This can be explained by the fact that participation fee (the fee for 2005 was € 1200) is quite high and more likely to be afforded by senior surgeons. However this cost only covers the overheads of the course. This highlights the difficulties in participation of post-graduate residents that are still in training, unlike the American course that attracts not only attending surgeons but also fellows and residents [9]. One way to attract the young surgeons to this valuable course would be if it became recognized as an integral part of the residency.

Ideally, a course should provide a theoretical education and a practical training of participants, by obtaining their direct involvement, and thus responding to their needs and expectations. With regards to the continuous education program, a future improvement of the course might be achieved by organizing workshops on particular clinical cases or on particular implications in the treatment of polytrauma. We are currently preparing a questionnaire to distribute to ex-participants in order to verify the course's impact on their day-to-day work.

The problem of training trauma surgeons, i.e. the lack of experience in treating traumas in operating room, is common to all countries [16] and the education programs proposed are substantially similar in content and practical approach (the use of animal models and the modality of the surgical scenarios); because all trauma surgeons mainly treat thoraco-abdominal injuries, except for the surgeons practicing in German speaking countries, who usually treat even orthopedic and neurosurgery traumas [5].

Since we are aware that most participants of our courses work in hospitals where the management of trauma patients is not common, we have introduced sections focusing on diagnosis, the emergency approach, and nursing management in the operating room and in the ward. This represents the peculiarity of our course compared to others.

Finally, considering that most training programs have a common basis and teaching method, we think that cooperation among the teaching staffs that organize similar courses would be useful to ensure a uniform standard course with a single tested method with regards to evaluation of the participants, choice and assessment of the teaching staff, and planning of updating. This could lead to obtaining official approval as already occurs for the ATLS and, therefore, provide all participants with a similar background.

## Conclusion

In an age of advanced technology in distance learning with telematic and computer methods, we believe that a course on the management of trauma, designed to create extremely realistic conditions of stress in the operating room as in life-like situations is very useful to train the trauma surgeon.

Besides the efficacy of the course, many other aspects should be discussed in the training institutions. For instance, if these courses are organized especially for surgeons who sporadically run into the traumatic diseases, should they become a mandatory part of training the general surgeon? If the main goal is to enable all surgeons to deal with traumas in the best possible way, how can we motivate students-in-training, who can hardly bear the expenses? On the basis of the results obtained previously in our courses and those of similar education programs, we can conclude that a theoretical and practical course, such as the "Trauma Surgery Course", is a good updating tool on trauma pathology for surgeons who work in Hospital Emergency Surgery Units or those belonging to Humanitarian Organizations, who are used to dealing with this pathology in foreign countries. Skills in trauma surgery should be an integral part of the surgeon's training and it would be important to gain the support of Scientific Societies (the Italian Society Emergency and Trauma Surgery has already expressed an interest in this education program) to carry out these courses. Integration with other courses would lead to a wider diffusion and recognition of the teaching method.
